# Investigating the Dosimetric Impact of Acuros XB in Lung Cases Before Clinical Implementation

**DOI:** 10.7759/cureus.93913

**Published:** 2025-10-06

**Authors:** Marina Chalkia, George Patatoukas, Maria Tsimpoukelli, Nikolaos Kollaros, Maria-Eleni Zachou, Efrosyni Kypraiou, Vassilis Kouloulias, Kalliopi Platoni

**Affiliations:** 1 Department of Applied Medical Physics, Attikon University Hospital, Athens, GRC; 2 Department of Clinical Radiation Oncology, Attikon University Hospital, Athens, GRC; 3 Department of Clinical Radiation Oncology, National and Kapodistrian University of Athens, Medical School, Attikon University Hospital, Athens, GRC; 4 Department of Applied Medical Physics, National and Kapodistrian University of Athens, Medical School, Attikon University Hospital, Athens, GRC

**Keywords:** aaa, acuros xb, dosimetric comparison, lung cancer, vmat

## Abstract

Background

This study aims to present a simple and reliable way to study the dosimetric impact of the Acuros XB (AXB) algorithm compared to the Anisotropic Analytical Algorithm (AAA). Volumetric modulated arc therapy (VMAT) plans for lung cancer treatments were studied when changing the planning target volume (PTV) margins, positions, and interface distances.

Methodology

Three PTV positions were studied: near an air/tissue interface (PTVint), at the upper lobe surrounded by air (PTVair), and at the mediastinum (PTVtis). For the PTV near the interface, four VMAT plans were created, expanding the PTV margin and reducing the air/tissue interface distance at the same time. The margins were set to 0.5 cm, 0.7 cm, 1 cm, and 1.5 cm from the gross target volume (GTV). Differences in dose distributions between AXB and AAA occurred in all PTVs, with the maximum differences seen in PTVs including more air volume.

Results

For PTVtis, the two algorithms presented similar behavior. Specifically, for PTV mean dose (D_mean_), AXB showed lower values of -2.3%, while for PTVair and PTVint, the mean corresponding differences were -18.2% and -10.1%. Concerning the mean lung doses (MLDs), AXB showed lower MLD than AAA for most PTVs, with differences ranging from -5.5% to -13.2%. For the expanding PTVs, MLD increased similarly for the two algorithms, i.e., 2.5 Gy to 4.9 Gy for AAA and 2.2 Gy to 4.4 Gy for AXB (for margins 0.5 cm to 1.5 cm). AAA overestimated the dose to the spine for most PTVs, with mean differences ranging from 4.7% to 15%. Conformity (CI) and Homogeneity indices (HI) also presented differences, with AAA plans showing higher PTV conformity, i.e., CI near unity, while CI values of AXB plans ranged from 0.27 to 0.94. Additionally, AAA plans were more homogeneous, with HI values ranging from 7.0 to 9.6, while HI values of AXB plans ranged from 19.1 to 24.9.

Conclusions

For lung cancer treatments, the PTV position, PTV margin, interface distance, and algorithm implemented can affect the dose calculation accuracy. Therefore, attention is required to avoid PTV underdosing or normal tissue toxicity. The simple-to-apply method presented here could be clinically applied and proven useful for that analysis. The two main limitations of the present study are the limited sample size and the fact that all plans were recalculated with AXB, keeping the same optimization objectives as AAA. Thus, the results should be interpreted with caution in clinical application.

## Introduction

Lung cancer is the leading cause of cancer-estimated deaths in the United States for both men and women, with approximately 230,000 new invasive cases diagnosed in 2022 [1]. For lung cancer patients, the current standard of care is a combination of radiotherapy and chemotherapy [2,3].

Along with surgery, chemotherapy, and immunotherapy with biomarkers, radiation therapy (RT) is an important component of cancer treatment, with approximately 50% of all cancer patients receiving RT during their course of illness [4,5]. In recent years, ongoing advances in RT techniques have attempted to increase the curative effect and reduce the side effects for cancer patients. Developments in this field include advances in imaging techniques, motion management, treatment planning systems, and radiation treatment modalities, which can deliver high doses of radiation with high conformity. Intensity modulated radiation therapy (IMRT) and volumetric modulated arc therapy (VMAT) are modern techniques that achieve high dose conformity to the tumor while sparing normal tissue. Although three-dimensional conformal radiotherapy (3D-CRT) remains a minimum standard option for non-small-cell lung cancer (NSCLC), the more advanced RT techniques, IMRT and VMAT, have been shown to be able to increase the prescription dose with comparable toxicity profiles with 3D-CRT [1].

With the development of RT techniques, there is an ongoing demand for highly accurate dose calculation algorithms. Specifically, in lung cancer, where dose escalation, high risk of lung toxicity, and tissue heterogeneity are involved, precise and accurate dose calculation methods should be applied. The algorithms used in treatment planning systems may fall into one of the following categories: correction-based, model-based, or Monte Carlo (MC). Each category has advantages or disadvantages when used for different dose calculations. MC methods are broadly considered the gold standard for accurate dose calculation in RT, as they can overcome the limitations of simpler algorithms, improving their systematic tumor dose overestimation and lung dose underestimation [2]. In the past decades, researchers considered that MC-based methods would not be used for routine treatment planning due to the long calculation times. Nevertheless, their introduction into the clinical practice is ongoing, and several treatment planning system (TPS) vendors offer MC dose calculation engines [6]. Pencil beam (PB) is a correction-based algorithm and is the most widespread photon dose calculation algorithm until recently [2]. It performs calculations on an infinitely narrow pencil beam dose distribution, taking into account the inhomogeneity correction for the longitudinal direction in the central beam axis, ignoring lateral scatter [3]. This restriction makes PB inadequate to properly compute dose distributions in lung cancer treatments, where tissue heterogeneities prevail.

More advanced algorithms accounting for lateral electron transport have been developed, such as the Anisotropic Analytical Algorithm (AAA). The AAA is a model-based algorithm, introduced in the Eclipse (Varian Medical Systems) treatment planning system to replace the PB algorithm, as it improves the accuracy of dose calculations [7]. The total dose deposition is calculated as the superposition of the MC-derived dose kernels of both primary and scattered components. However, the tissue inhomogeneity is corrected by simplified density scaling of the kernels, such that the secondary electron transport is only modeled macroscopically [8]. Studies have shown that AAA overestimates the dose near air-tissue interfaces and the near-minimum target dose, leading to a lower target coverage than the prescribed dose [1,9].

A new model-based algorithm has been investigated and applied to clinical practice, Acuros XB (AXB) [10,11]. AXB solves with numeric methods the linear Boltzmann transport equation and can achieve accurate modeling of dose deposition in heterogeneous media comparable to that of MC-based methods [2,12].

In our department, we use the Varian Eclipse software version 17.1 TPS with the AAA for dose calculations, and quite recently, we introduced the AXB algorithm. Before the implementation of the new algorithm in clinical routine, we investigated the dosimetric impact of the two algorithms in VMAT plans for lung cancer treatments under different conditions. Due to the heavy workload of the department, a simple-to-apply method was chosen instead of a more analytical one. First, we wanted to investigate the dosimetric behavior of each algorithm for different planning target volume (PTV) positions inside the lung. Second, we evaluated the dosimetric differences when changing the PTV margins (including more or less air) and interface distances. For the treatment planning of lung cancer treatment under free breathing, the end-of-exhale and end-of-inhale breath-hold images were used to set an internal target volume (ITV). An additional margin, e.g., the PTV, was delineated to encompass the whole respiratory tumor motion area and to account for other treatment uncertainties such as setup or delineation uncertainties. Thus, we studied the impact of four different PTV margins. Lastly, we wanted to study the assumption that AAA underestimates the dose to the spinal cord [13].

## Materials and methods

In our study, CT images from 17 patients, 10 males and 7 females, were included. The study was conducted in the Department of Radiotherapy, Attikon University Hospital in Athens, Greece. The study sample was selected based on patient sex to gather data from both male and female patients, on PTV volume, ranging from 15 cc to 25 cc, and on PTV position. PTVs positioned in the right upper lung lobe, in the mediastinum, and near an air/tissue interface were included. VMAT plans for these 17 lung cancer patients were generated using 6 MV photon beams. All calculations were performed with the TPS Eclipse version 17.1 with dose calculation algorithms AAA and AXB. The calculation grid of 2.5 mm was used. For the CT-sim scanning, a matrix size of 642 × 642 pixels for a field of view of 57 × 57 cm^2^ was used, and the slice thickness was set to 3 mm. For the commissioning process and accuracy of AXB, our protocol included TPS calculations of point doses with a virtual water-tank setup. The comparison with the water-tank measurements was conducted using an ionization chamber. Subsequently, a wide variety of clinical plans were studied for comparison with AAA and validated using an appropriate patient-specific quality assurance phantom (ArcCHECK, Sun Nuclear Corporation).

**Figure 1:**
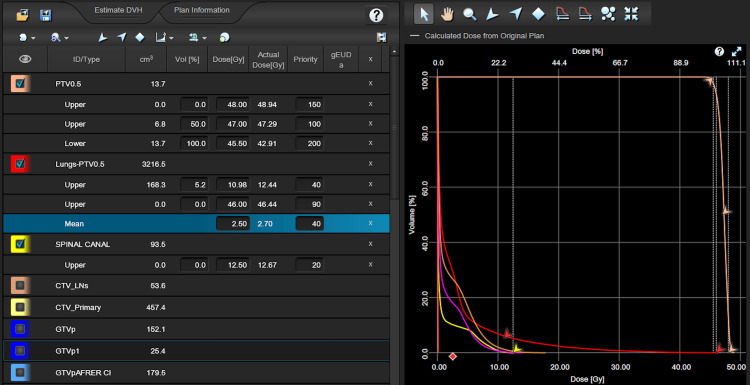
Optimization objectives used for the planning target volume and organs at risk (Varian Eclipse treatment planning system v.17.1).

A hypofractionated radiotherapy dose scheme of 45 Gy was applied, namely, 15 fractions of 3 Gy each. The plans were developed using one full arc with AAA first and then re-optimized and calculated with the AXB algorithm, keeping the same optimization objectives. The optimization objectives included the PTV, lungs, and spinal canal. The lung as an organ at risk (OAR) was delineated as lungs minus PTV. The heart was not included in the optimized OARs as the PTVs were not in close contact with the heart. Figure 1 shows the optimization objectives used for the PTV and OARs. Analysis was carried out with dose-volume histograms (DVHs) with the relative differences between each metric in the AAA and AXB plans for two different situations analyzed in the next sections. The monitor units of each plan were also recorded for comparison and evaluation.

Statistical analysis was performed using the t-test in the RStudio software (Developer: Posit, PBC, Boston, Massachusetts, USA. Version: RStudio 2023.09.1+494 “Desert Sunflower”. Packages used: readxl, ggplot2) to assess the statistical significance of the differences with a significance level set at p-values <0.05.

PTVs in different lung positions

**Figure 2:**
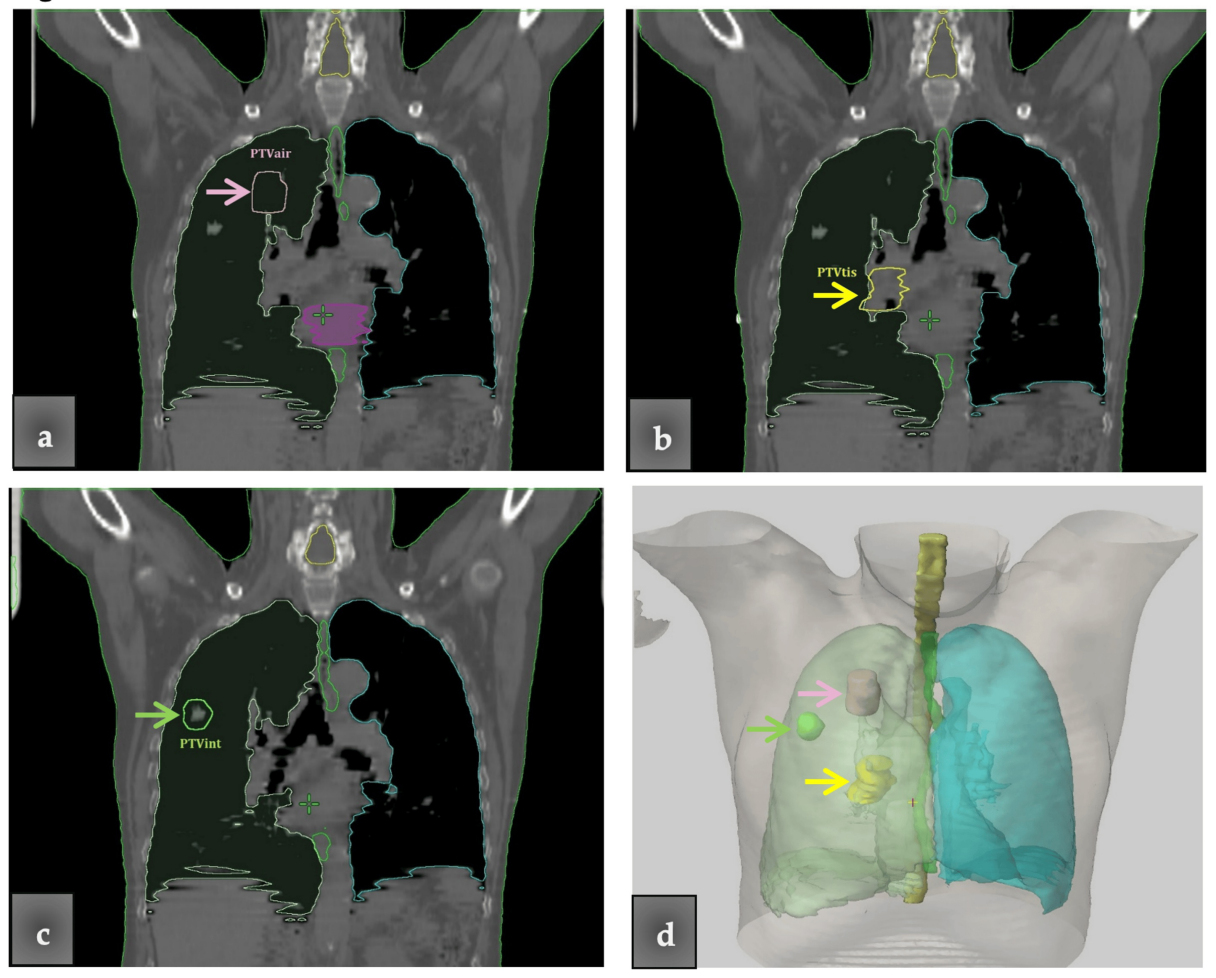
Three planning target volumes (PTVs) located in three different locations in the right lung. (a) The pink arrow indicates the PTVair, including mostly air volume, and surrounded by air. (b) The yellow arrow indicates the PTVtis positioned in the mediastinum and includes mostly lung tissue. (c) The green arrow indicates the PTVint positioned near an air/tissue interface and including the gross tumor volume (GTV). (d) All three PTVs (indicated by the arrows) are shown in the three-dimensional display.

Three PTVs were delineated, each one representing a different target and surrounding tissue situation. The PTVs were positioned at the left and right lung, and the study cohort included both male and female patients to cover a variety of possible PTV positions and patient anatomy. The first one, called PTVair, was positioned in the upper lung lobe; it included mostly air volume and was surrounded by air (Figure 2a). The second one, called PTVtis, was positioned mostly in the mediastinum, and the biggest part of its volume included lung tissue, e.g., the cancer site (Figure 2b). The third one, called PTVint, was positioned near an air/tissue interface (Figure 2c). All three PTVs are displayed for one patient in a three-dimensional display in Figure 2d. The relative differences of the dosimetric parameters were calculated with Equation 1:

\begin{equation} \text{Relative difference (\%)} = \frac{\text{AXB value} - \text{AAA value}}{\text{AAA value}} \cdot 100 \label{eq:relative_difference} \end{equation}

For all the PTVs, the dosimetric parameters evaluated were maximum, mean, and minimum doses, and the conformity index (CI). The CI represents the relationship between isodose curves and target volume [14]. A CI near unity indicates high PTV coverage and low irradiation of surrounding tissues. The CI was calculated according to the definition of the ICRU 62, which is the quotient of the treated volume (TV) and the PTV (Equation 2) [15]. TV is the volume specified by the radiation oncologist as being appropriate to receive the prescribed dose, which in our case is the volume enclosed by the 95% isodose curve.

\begin{equation} CI = \frac{TV}{V_{\text{PTV}}} \label{eq:ci_equation} \end{equation}

where TV is the treated volume and V_[PTV] is the PTV volume.

For the lungs, the dosimetric parameters evaluated were the mean lung dose (MLD) and V20 (lung volume minus PTV receiving 20 Gy or more). For the spinal canal, the maximum dose was recorded (D_1%_). The MUs were also recorded for each PTV.

Changing PTV margins and interface distances

**Figure 3:**
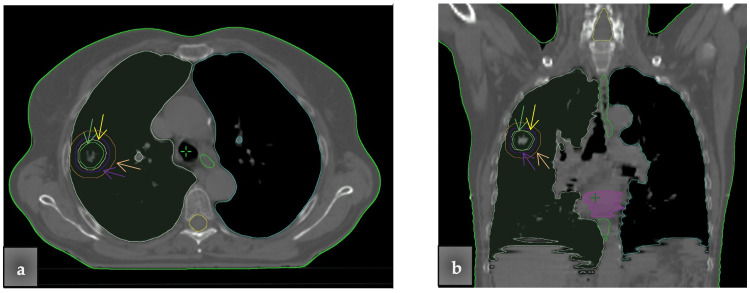
Four different planning target volume (PTV) margins from the gross target volume (GTV) were delineated. The PTV margins were set to 0.5 cm (green line and arrow), 0.7 cm (yellow line and arrow), 1 cm (purple line and arrow) and 1.5 cm (orange line and arrow), while the interface distances were 1 cm, 0.8 cm, 0.5 cm, and 0 cm, correspondingly. (a) Transversal view. (b) Coronal view.

For the PTVint, four different PTV margins from the gross target volume (GTV) were used for the dosimetric evaluation of the two algorithms (Figure 3). Specifically, the PTV margins were set to 0.5 cm, 0.7 cm, 1 cm, and 1.5 cm, creating the PTV 0.5, PTV 0.7, PTV 1, and PTV 1.5, correspondingly. These specific margins were chosen to correspond to the clinical ones. According to RTOG 0617, a CTV margin of 0.5-1 cm, and a PTV margin of even 1.5 cm in the absence of motion management technique is required [16]. The smaller PTV margins (e.g., 0.5 cm) are used in stereotactic body radiation therapy (SBRT) to protect the OARs from the higher PTV doses delivered. As the PTV margins were expanded, the air/tissue interface distance was reduced.

Except for the dosimetric parameters mentioned above, the homogeneity index (HI) was also calculated. HI is an objective tool to analyze the uniformity of dose distribution in the PTV [17]. Various formulae have been used for calculation, but we chose the descriptive formula seen in Equation 3. This formula uses the near-maximum (D2) and near-minimum dose (D98) instead of true maximum or minimum doses, which are sensitive to several parameters, such as grid size and high dose gradient [17,18].

\begin{equation} HI = \frac{D_2 - D_{98}}{D_P} \cdot 100 \label{eq:hi_calculation} \end{equation}

where D_2_ is the dose to 2% of the target volume (near-maximum dose), D_98_ is the dose to 98% of the target volume (near-minimum dose), and D_P_ is the prescribed dose.

## Results

PTVs in different lung positions

**Figure 4:**
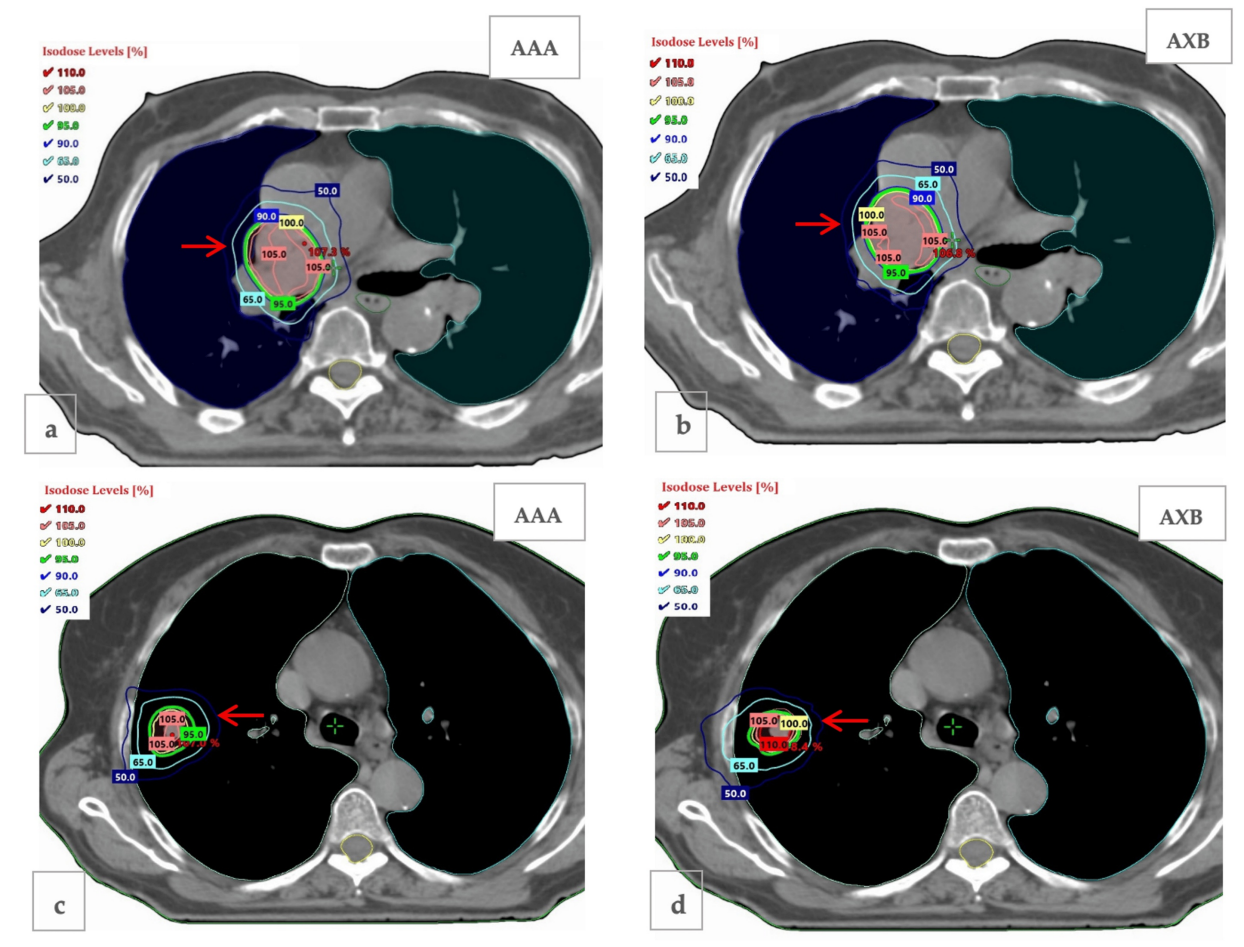
The red arrows indicate the isodose curves at axial views for (a) PTVtis calculated with anisotropic analytical algorithm (AAA), (b) PTVtis calculated with Acuros XB (AXB), (c) PTVint calculated with AAA, and (d) PTVint calculated with AXB. PTV: planning target volume

Figure 4 shows the isodose curves at axial views for PTVtis and PTVint given by the two algorithms for a patient. For PTVtis, the isodose curves show similar behavior, while for PTVint, they seem to extend differently toward the tissue beyond the interface, with 65% and 50% isodoses covering part of the tissue and rib bone. Dosimetric analysis of the plans provided the DVH comparisons of PTV and OAR doses between the two algorithms. Differences in dose distributions between AXB and AAA occurred in all three PTVs, with the maximum differences seen in PTVair. For the PTVtis, small differences were found for the PTV D_max_ and mean doses (D_mean_).

**Figure 5:**
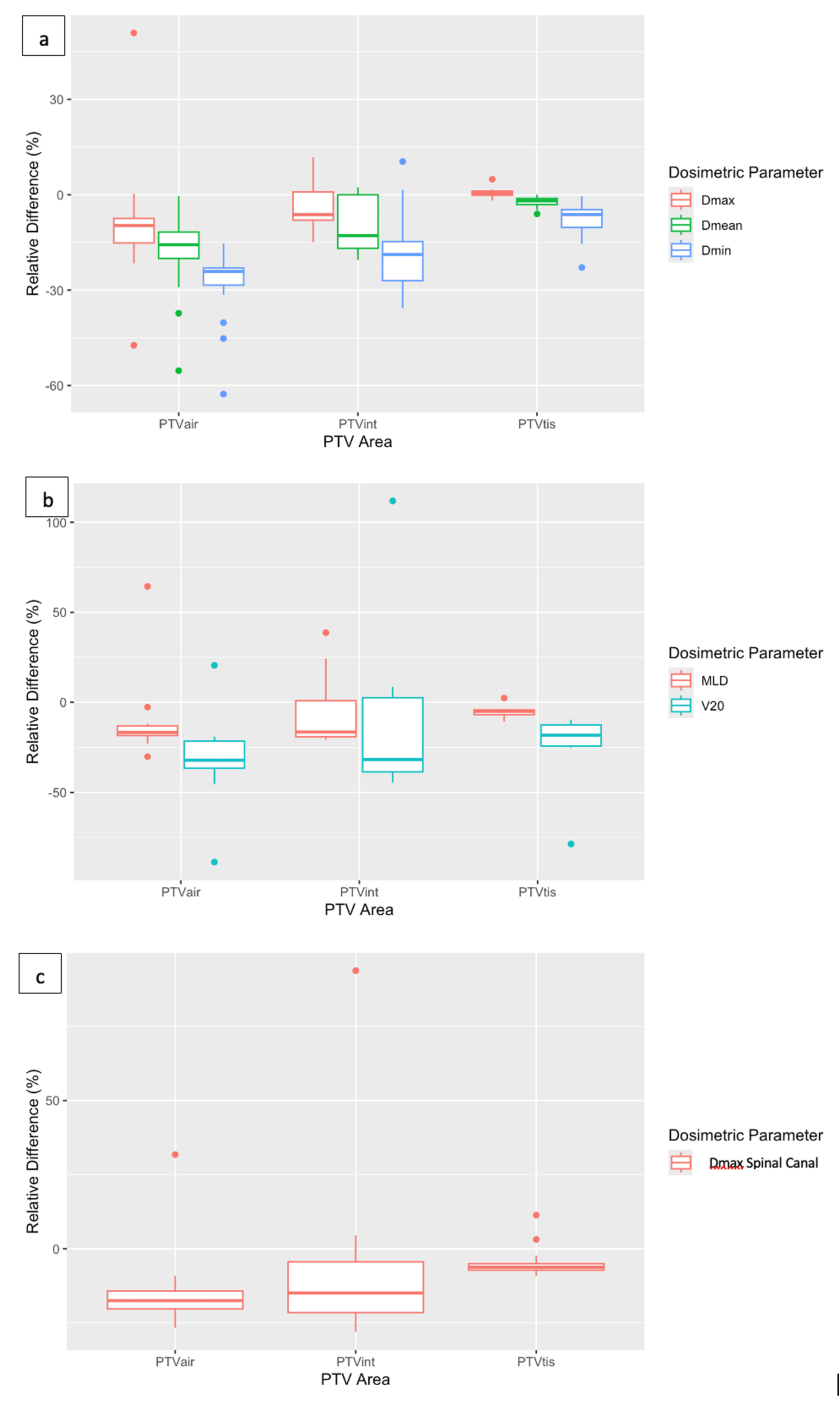
The relative difference between anisotropic analytical algorithm (AAA) and Acuros XB (AXB) for the three different planning target volumes (PTVs) delineated in the lung, e.g., PTVair includes air volume and is surrounded by air, PTVtis includes lung tissue, and PTVint includes air and tissue and is close to the air/lung interface, for (a) total maximum dose (Dmax), PTV maximum dose and PTV mean dose (Dmean); (b) mean lung dose (MLD) and V20 (the lung volume receiving 20 Gy); (c) spinal canal maximum dose (D1%).

Analysis of the DVHs led to the relative differences between AXB and AAA (Figure 5). The relative differences were calculated with Equation 1. PTVair showed the largest relative differences for D_max_, D_mean_, and D_min_, namely, -9.6%, -18.2% and -27.9% mean values, respectively. PTVtis, on the other hand, showed the smallest relative differences, namely, 0.7%, -2.3%, and -7.7% mean values, correspondingly. Statistical analysis of these dosimetric values for all three PTVs showed that all relative differences, except for D_max_ of PTVair and PTVtis, were statistically significant (Table 1).

**Table 1:** Relative differences (mean and standard deviation (SD) values) between Anisotropic Analytical Algorithm (AAA) and Acuros XB (AXB) for the planning target volumes (PTVs) in three different lung positions, concerning PTV Dmax, Dmean and Dmin, lung mean lung dose (MLD), and V20, and spinal canal D1%. The p-values and t-values derived from the statistical analysis are also presented. Statistical test used: one-sample t-test.

Volume	Dosimetric parameter	Relative difference (AXB-AAA)/AAA × 100%, mean (SD)	P-value	Statistically significant (yes/no)	t-value (one-sample t-test)
PTV					
PTVair	D_max_	-9.6 (18.7)	0.051	No	-2.11
	D_mean_	-18.2 (13.0)	<0.001	Yes	-5.77
	D_min_	-27.9 (11.7)	<0.001	Yes	-9.85
PTVint	D_max_	-4 (6.4)	0.020	Yes	-2.56
	D_mean_	-10.1 (8.4)	<0.001	Yes	-4.94
	D_min_	-17.0 (13.5)	<0.001	Yes	-5.22
PTVtis	D_max_	0.7 (1.4)	0.64	No	1.98
	D_mean_	-2.3 (1.6)	<0.001	Yes	-5.95
	D_min_	-7.7 (5.7)	<0.001	Yes	-5.53
Lungs (lungs minus PTV)					
PTVair	MLD	-11.7 (20.4)	0.03	Yes	-2.36
	V_20_	-30.7 (20.8)	<0.001	Yes	-6.08
PTVint	MLD	-8.1 (17.1)	0.7	No	-1.94
	V_20_	-16 (37.7)	0.1	No	-1.74
PTVtis	MLD	-5.5 (2.9)	<0.001	Yes	-7.84
	V_20_	-21.9 (16.6)	<0.001	Yes	-5.09
Spinal canal					
PTVair	D_1%_	-15 (12.9)	<0.001	Yes	-4.80
PTVint	D_1%_	-7.6 (27.7)	0.28	No	-1.12
PTVtis	D_1%_	-4.7 (5.0)	<0.001	Yes	-3.93

As far as the lungs are concerned, AXB showed lower mean lung doses and V_20_ than AAA. The highest difference of mean values was -30.7% for the V_20_ of PTVair, while the lowest one was -0.5% for the V_20_ of PTVtis (Figure 5b). For MLD, the PTVs showed the same behavior with V_20_ (i.e., the highest differences for PTVair and the lowest differences for PTVtis). Statistical analysis showed that lung dose differences were statistically significant for PTVair and partly for PTVtis (i.e., only for MLD) but showed no significance for PTVint (Table 1). Dose differences were also found at the level of the spinal canal, especially for the PTVair, where the mean relative difference of D_1%_ reached -15% (Figure 5c). It should be noted that PTVint values showed a large standard deviation of 27.7%. Statistical analysis showed again no statistically significant findings for PTVint (Table 1).

**Figure 6:**
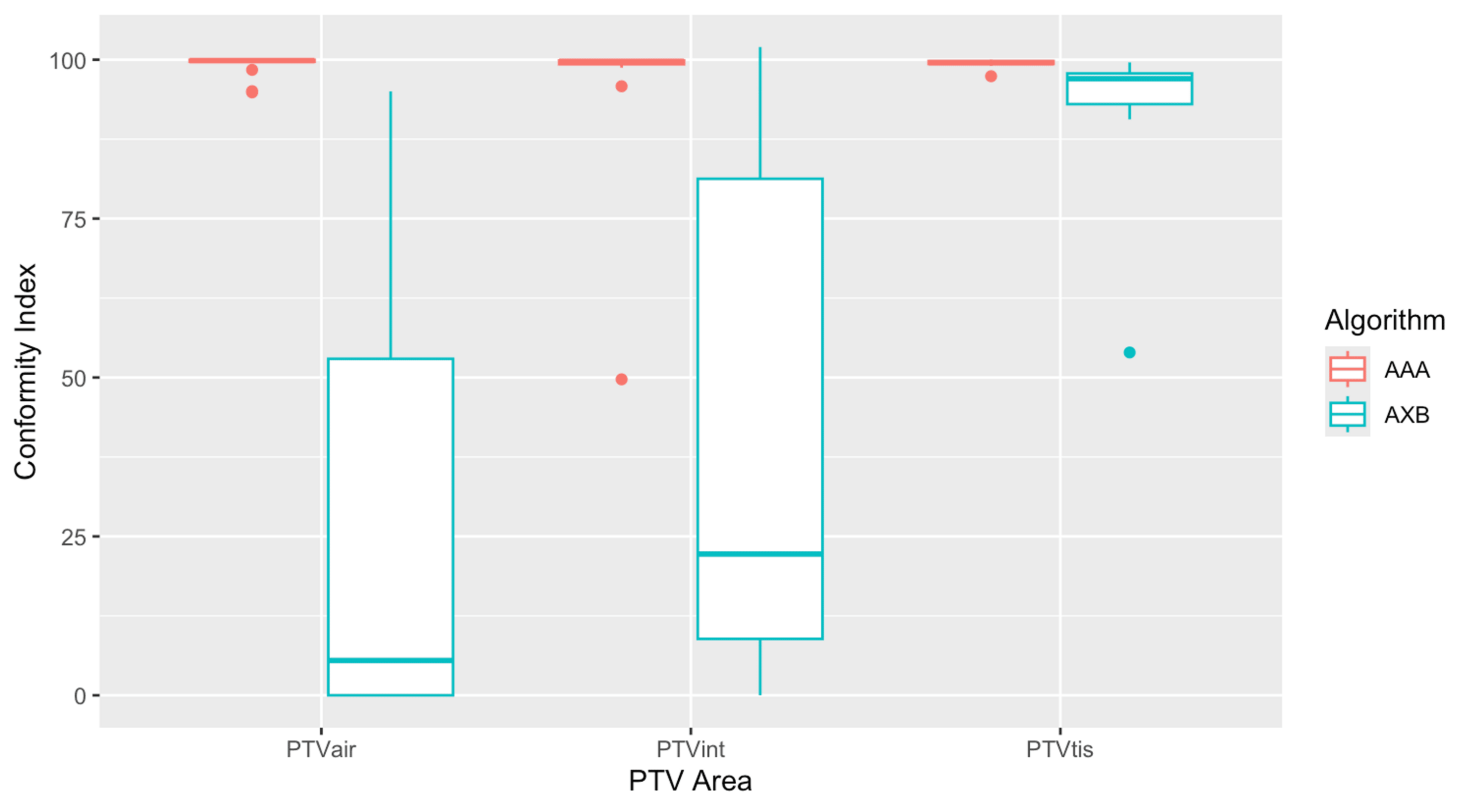
Conformity index (CI) for each plan of the three planning target volumes (PTVs) calculated with the Anisotropic Analytical Algorithm (AAA) and Acuros XB (AXB).

Figure 6 displays the box plots of the CI calculated for each plan of the three PTVs. The diagram shows the minimum, maximum, sample median, first and third quartile values, and any outliers of the data. AAA showed almost ideal CI (=1) for all PTVs, while AXB showed much lower CI values. The highest difference was noted for the PTVair, where AAA had an ideal mean CI of 0.99, while AXB had a much lower CI of 0.27 (Table 2). For the PTVtis, on the other hand, the algorithms presented similar behavior, with a CI near unity (CI = 0.99 for AAA and 0.94 for AXB). Statistical analysis showed that CI values for the two algorithms were statistically significant for all PTVs (Table 2).

**Table 2:** Conformity index (CI) calculated for each plan of the three planning target volumes (PTVs) (mean and standard deviation (SD) values), as well as the p-values and t-values from the statistical analysis of the two algorithms. Statistical test used: Welch two-sample t-test.

Volume	CI, mean (SD)		P-value	Statistically significant (yes/no)	t-value (Welch two-sample t-test)
	AAA	AXB			
PTVair	99.2 (1.6)	27.2 (33.4)	<0.001	Yes	8.89
PTVint	96.5 (12.1)	44.7 (38.8)	<0.001	Yes	5.25
PTVtis	99.4 (0.6)	93.6 (10.6)	0.03	Yes	2.28

**Figure 7:**
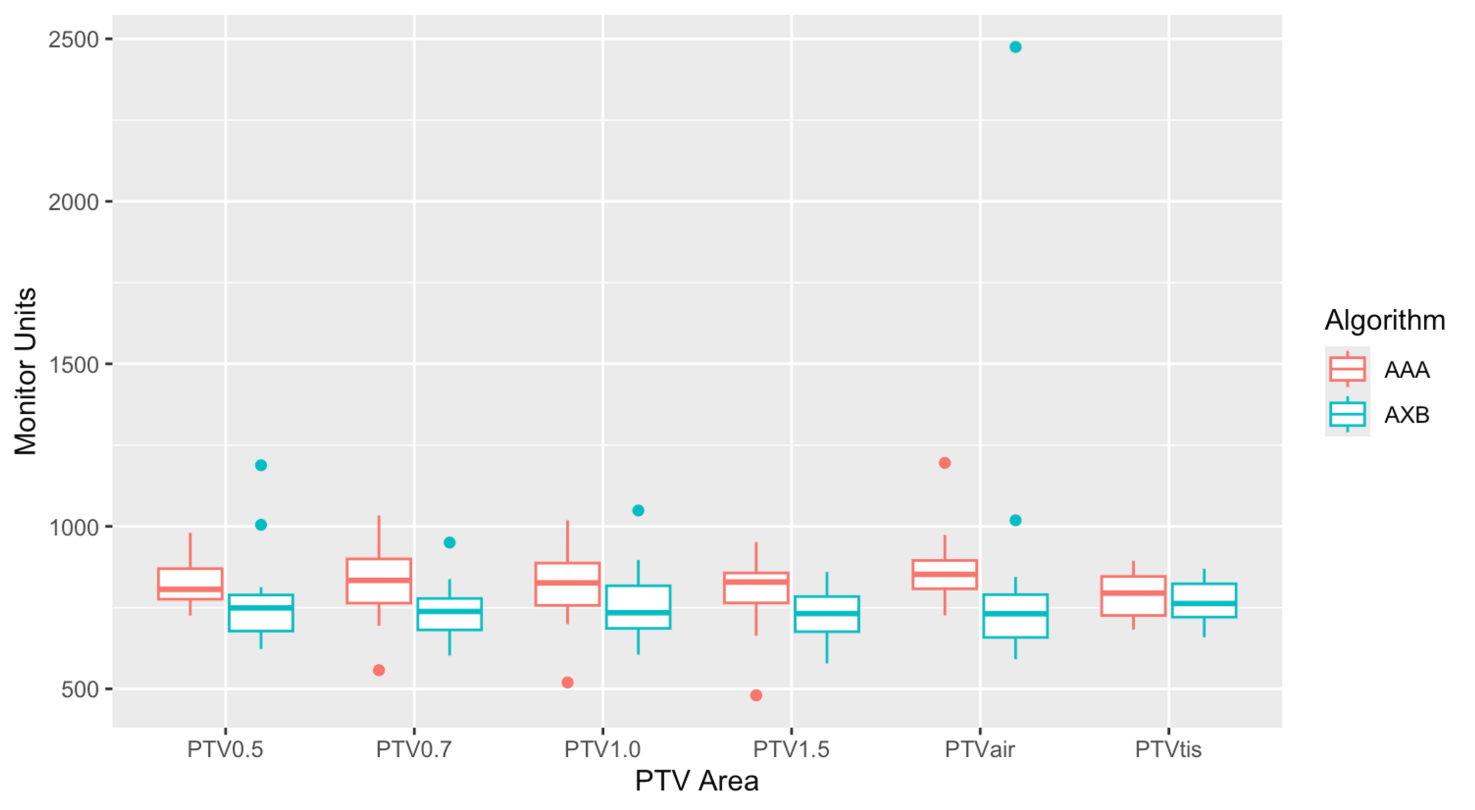
Box plots for the calculated monitor units (MUs) of all plans (where the planning target volume (PTV) 0.5 coincides with PTVint).

The monitor units (MUs) of each plan are summarized in Table 3. Figure 7 displays the box plots of the calculated MUs for all plans. Apart from a few outliers, AXB had fewer MUs for all plans, with PTVint having the largest difference (i.e., mean value difference of 50 MUs) and PTVtis the smallest (i.e., mean value difference of 19 MUs). However, the statistical analysis showed no statistical significance for either PTV (Table 3).

**Table 3:** Monitor units (MUs) of each plan for planning target volumes (PTVs) in different locations and for PTVs with different margins (mean and standard deviation (SD) values). P-values and t-values are also given for each correlation. PTVint is the same with PTV 0.5. Statistical test used: Welch two-sample t-test.

Volume	MUs, mean (SD)		P-value	Statistically significant (yes/no)	t-value (Welch two-sample t-test)
	AAA	AXB			
PTVair	863.8 (105)	837.7 (433.3)	0.8	No	0.24
PTVint (=PTV 0.5)	821.3 (69.4)	770.5 (139)	0.2	No	1.34
PTVtis	787.6 (69.6)	768.2 (67.6)	0.4	No	0.83
PTV0.7	827.3 (112.7)	737.6 (88.6)	0.015	Yes	2.58
PTV1	825.0 (119.1)	755.1 (111.6)	0.087	No	1.76
PTV1.5	796.0 (110.0)	720.2 (79.6)	0.028	Yes	2.30

Changing PTV margins and interface distances

**Figure 8:**
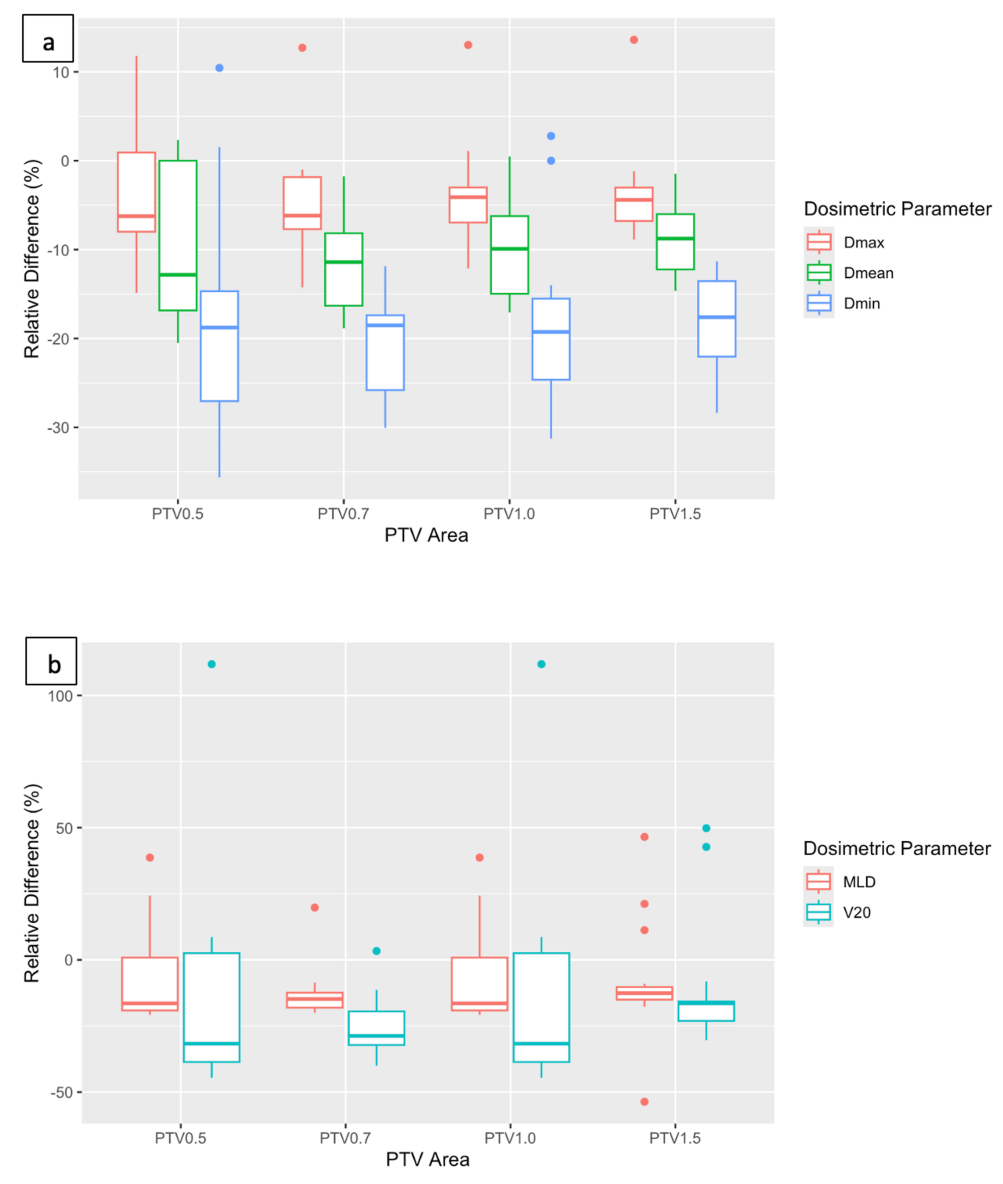
Relative difference between Anisotropic Analytical Algorithm (AAA) and Acuros XB (AXB) for the planning target volumes (PTVs) with four different margins from the gross target volume (GTV). The box plots show (a) the PTV maximum dose (Dmax) and PTV mean dose (Dmean); (b) mean lung dose (MLD) and V20 (the lung volume receiving 20 Gy).

For the VMAT plans with expanding GTV margins, the box plots of relative differences between AXB and AAA are presented in Figure 8. The PTV D_max_ was lower for AXB plans, with a few exceptions, and the mean relative differences were close to -4% (Table 4). AXB also showed lower values of PTV D_mean_ and D_min_ for all margins. The largest differences were observed at D_min_, where they reached -21% for PTV 0.7. Statistical analysis showed that the relative differences between the two algorithms for all four PTVs were statistically significant (Table 4).

**Table 4:** Relative differences (mean and standard deviation (SD) values) between Anisotropic Analytical Algorithm (AAA) and Acuros (AXB) for the planning target volumes (PTVs) with four different margins, concerning PTV Dmax, Dmean and Dmin, lung mean lung dose (MLD), V20, and spinal canal D1%. The p-values and t-values derived from the statistical analysis is also presented. Statistical test used: one sample t-test

Volume	Dosimetric parameter	Relative difference (AXB-AAA)/AAA × 100%, mean (SD)	P-value	Statistically significant (yes/no)	t-value (one-sample t-test)
PTV					
PTV 0.5	D_max_	-4 (6.4)	0.021	Yes	-2.56
	D_mean_	-10.1 (8.4)	<0.001	Yes	-4.94
	D_min_	-17 (13.5)	<0.001	Yes	-5.22
PTV 0.7	D_max_	-4.8 (5.7)	0.003	Yes	-3.44
	D_mean_	-11.6 (5.2)	<0.001	Yes	-9.27
	D_min_	-21.0 (5.8)	<0.001	Yes	-15.02
PTV 1	D_max_	-4 (5.5)	0.008	Yes	-3.00
	D_mean_	-9.8 (5.9)	<0.001	Yes	-6.83
	D_min_	-19.0 (9.3)	<0.001	Yes	-8.48
PTV 1.5	D_max_	-3.9 (5.1)	0.006	Yes	-3.14
	D_mean_	-9.0 (3.8)	<0.001	Yes	-9.83
	D_min_	-18.6 (4.9)	<0.001	Yes	-15.82
Lungs (lungs minus PTV)					
PTV 0.5	MLD	-8.1 (17.1)	0.07	No	-1.94
	V_20_	-16.0 (37.7)	0.1	No	-1.74
PTV 0.7	MLD	-13.2 (9.5)	<0.001	Yes	-5.59
	V_20_	-25.7 (11.3)	<0.001	Yes	-9.41
PTV 1	MLD	-11.2 (9.9)	<0.001	Yes	-4.63
	V_20_	-19.4 (16.2)	<0.001	Yes	-4.93
PTV 1.5	MLD	-8.5 (20.4)	0.11	No	-1.71
	V_20_	-11.9 (22.7)	0.046	Yes	-2.16
Spinal canal					
PTV 0.5	D_1%_	-7.6 (27.7)	0.28	No	-1.12
PTV 0.7	D_1%_	-12.9 (13.4)	0.001	Yes	-3.95
PTV 1	D_1%_	-10.9 (10.4)	<0.001	Yes	-4.33
PTV 1.5	D_1%_	-9.5 (12.9)	<0.001	Yes	-3.05

Analyzing the dose to the lungs, AXB had lower mean values for both the MLD and V_20_ for all margins (Figure 8b). PTV 0.5 had the lowest mean relative difference for MLD (-8.1%), while for V_20_, PTV 1.5 appeared to have the lowest mean relative difference (-11.9%) (Table 4). The relative differences for MLD were lower than those of V_20_ for all margins. Statistical analysis showed that lung relative differences for the two algorithms were statistically significant for all PTVs except for PTV 0.5 and for MLD of PTV 1.5 (Table 4). Table 5 shows the mean and standard deviation values for the MLD, V_20_, and D_1%_ of spinal canal for each algorithm separately. It can be noticed that expanding the PTV margin, the dose to both OARs became higher for both algorithms.

**Table 5:** Mean and standard deviation (SD) values for the MLD, V20, and D1% of spinal canal for each PTV margin and each algorithm separately. PTV: planning target volume; AAA: Anisotropic Analytical Algorithm; AXB: Acuros XB

PTVs	MLD (Gy), mean (SD)		V_20_ (%), mean (SD)		D_1%_ (Gy), mean (SD)	
Margin	AAA	AXB	AAA	AXB	AAA	AXB
PTV 0.5	2.5 (0.4)	2.2 (0.4)	2.1 (0.6)	1.7 (0.6)	9.1 (2.2)	8.1 (1.6)
PTV 0.7	3.0 (0.7)	2.7 (0.5)	3.2 (0.9)	2.4 (0.8)	10.6 (2.6)	9.1 (2.0)
PTV 1	3.9 (0.7)	3.4 (0.7)	4.4 (1.5)	3.5 (1.3)	12.4 (2.5)	10.9 (1.7)
PTV 1.5	4.9 (0.9)	4.4 (0.9)	6.5 (2.0)	5.5 (1.6)	15.2 (3.1)	13.5 (2.4)

**Figure 9:**
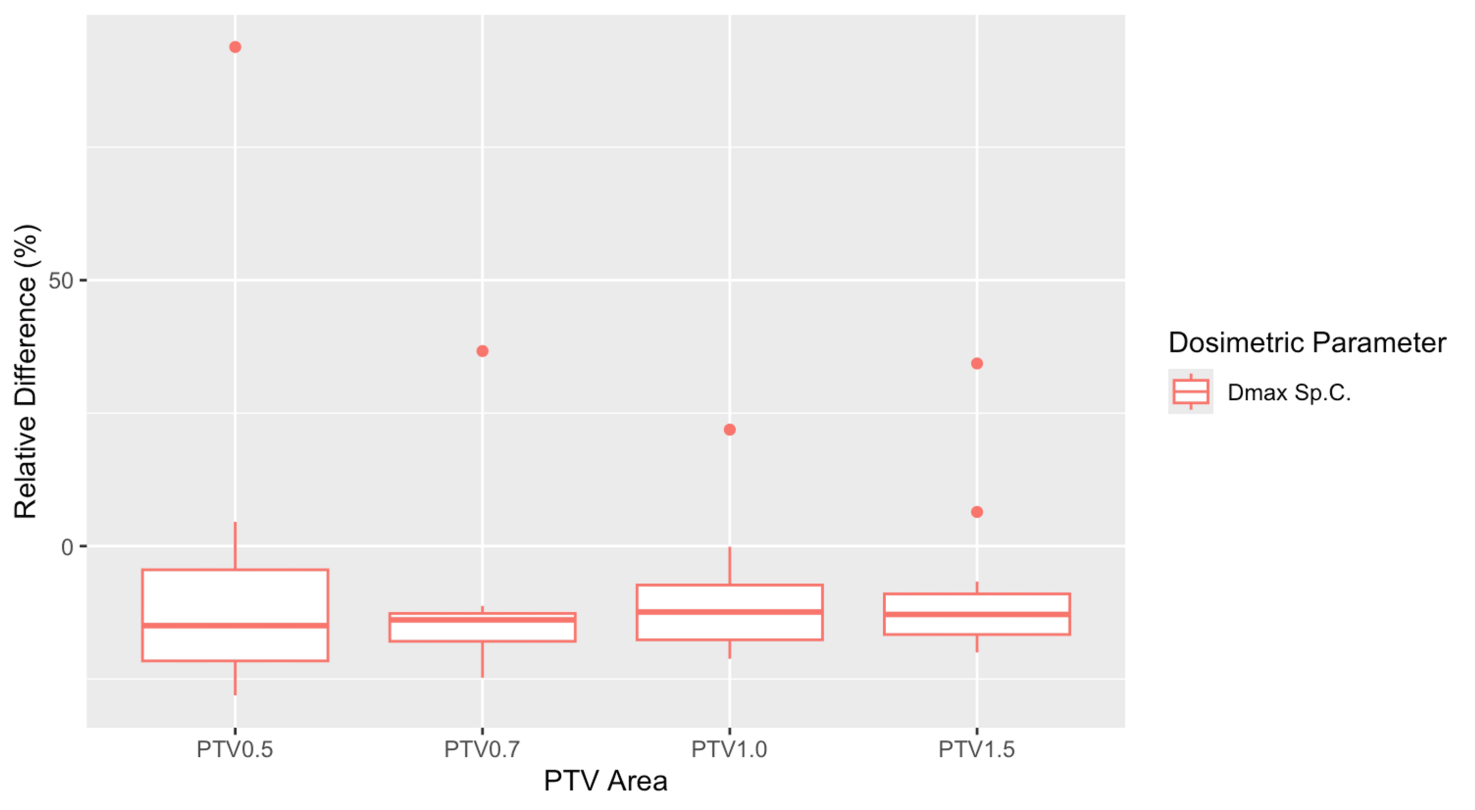
Box plots of the relative differences between Anisotropic Analytical Algorithm (AAA) and Acuros XB (AXB) plans for the maximum dose in the spinal canal (Dmax Sp.C.), calculated for each of the four different planning target volumes (PTVs) margins.

The dose calculations showed that AAA overestimated the dose to the spine for all four margins. The largest difference appeared to be for PTV 0.7, where the mean relative difference was -12.9%. Figure 9 shows the box plots of these differences for all PTV margins. Statistical analysis showed that spine relative differences for the two algorithms were statistically significant for all margins except for PTV 0.5 (Table 4).

**Figure 10:**
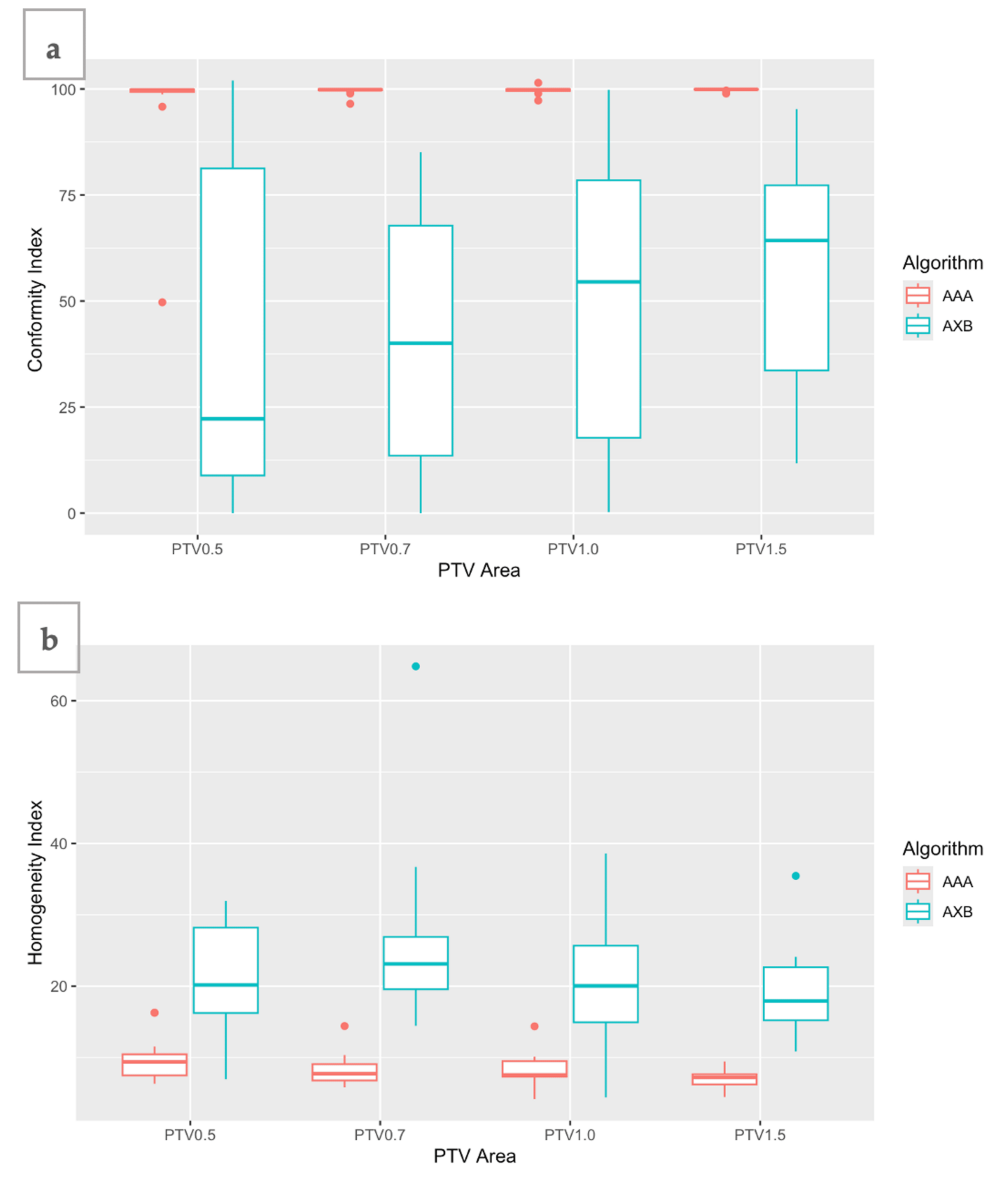
For each plan of the expanded planning target volumes (PTVs) calculated with Anisotropic Analytical Algorithm (AAA) and Acuros XB (AXB), differences in (a) the conformity index (CI) and (b) homogeneity index (HI) are shown.

Figure 10a shows the box plots of CI calculated for each plan of the expanded PTVs. All the AAA plans showed an ideal CI close to 0.99, while AXB plans showed a much lower CI mean value of 0.42-0.57. The PTV 1.5 plans were the most conformal ones. The HI for each plan is shown in Figure 10b. AAA plans were more homogeneous (mean HI = 7.0-9.6), while AXB plans had HI values higher than 19 (mean HI = 19.1-24.9). According to Equation 3, the minimum value of HI denotes the best homogeneity. Statistical analysis showed that CI and HI values for the two algorithms were statistically significant for all PTVs (Table 6).

**Table 6:** Conformity Index (CI) and Homogeneity Index (HI) calculated for each plan of the four PTV margins (mean and standard deviation (SD) values), as well as the p-values and t-values from the statistical analysis of the two algorithms. Statistical test used: Welch two-sample t-test. PTV: planning target volume; CI: conformity index; HI: homogeneity index; AAA: Anisotropic Analytical Algorithm; AXB: Acuros XB

PTVs	CI, mean (SD)		HI, mean (SD)		P-value	Statistically significant (yes/no)	t-value (CI)	t-value (HI)
Margin	AAA	AXB	AAA	AXB				
PTV 0.5	96.48 (12.1)	44.7 (38.8)	9.6 (3.0)	20.9 (7.5)	<0.001	Yes	5.25	-5.82
PTV 0.7	99.57 (0.8)	41.6 (30)	8.2 (2.1)	24.9 (11.8)	<0.001	Yes	7.96	-5.78
PTV 1	99.66 (0.8)	48.9 (34.6)	8.1 (2.3)	20.2 (8.1)	<0.001	Yes	6.06	-5.93
PTV 1.5	99.81 (0.3)	57.2 (27.6)	7.0 (1.3)	19.1 (5.8)	<0.001	Yes	6.37	-8.34

The MUs were also studied for all PTV margins. The MUs obtained from each plan are summarized in Table 3, while Figure 7 displays the box plots of the calculated MUs for all plans. Apart from a few outliers, AXB had fewer MUs for all plans, with PTV 0.7 having the largest difference (i.e., mean value difference of 90 MUs) and PTV 0.5 the smallest (i.e., mean value difference of 51 MUs). Statistically significant differences were found only for PTV 0.7 and PTV 1.5 (Table 3).

## Discussion

To our knowledge, the dosimetric analysis of different and well-defined target positions with a simple-to-apply method has never been published before. The PTV dose coverage presented large variations for the three different target locations and the different PTV margins. The differences in lung doses calculated by AAA and AXB, i.e., MLD and V_20_, were also dependent on the location of the target inside the lung and the density of the target. The spinal canal study showed that AAA predicted higher maximum doses. These results can indicate the most proper algorithm selected by the radiotherapy planners for treatment planning calculations. Having explicitly categorized the lung PTV positions in this study, one can trust either AXB or AAA and not exclude AAA from all lung cases. Furthermore, studying multiple PTV margins provides some preliminary results for the proper algorithm selection, as well as the margin selection based on the dosimetric results of each margin.

The photon dose calculation algorithms should present high calculation accuracy. This should be within a limit of 3%, taking into account the 5% limit recommended by the International Commission on Radiation Units and Measurements (ICRU) [19], and all other sources of uncertainty, such as patient setup, motion management, and machine calibration. This report is not intended to evaluate the clinical quality of the plans but to study the dosimetric impact and the calculation accuracy of the two algorithms when changing field sizes and the location of the target inside the lung. Under this scope, we chose to keep the optimization process for the AXB plans the same as the AAA plans and study the differences of AXB under the same objectives.

The three target locations had a different dosimetric impact on the PTV dose coverage. The highest differences for PTV D_max_, D_min_, and D_mean_ were seen in PTVair and PTVint, where PTV encompassed much air volume (PTVair) or was near an air-tissue interface (PTVint). The much lower density of the lungs, compared to tissue, resulted in higher differences in the two dose calculation algorithms. This was due to the increased electron interaction paths and the modeling of each algorithm for the lung heterogeneities. Taking into account the superiority of AXB in dose calculations for heterogeneous media [10,20,21], we can conclude that AAA estimated a lower dose deposited to PTV. The clinical impact of that possible tumor underdosage remains to be investigated. In PTVair, the mean relative differences were large, especially for D_min_. This can be attributed to the fact that PTVair included mainly air volume, and the algorithms tried to work on the objective D_95_ of 95% for a very low-density medium, having a different modelling of dose deposition in air. Thus, the dose calculated with AXB was more heterogeneous, introducing a significant decrease in the minimum PTV dose. It is an experimental PTV, not reflecting a clinical situation. Several other studies also reported lower values with AXB for D_min_ and D_mean_, while for D_max_, they found higher doses with AXB [1,2,22,23]. In PTVtis, the two algorithms presented a similar behavior. Differences in D_max_ and D_mean_ were below 3%, while D_min_ had a mean difference of -7.7%. This confirms the fact that AAA has high calculation accuracy in targets with density in the soft tissue range [10].

Dose calculated with AXB is more heterogeneous, as denoted by the quality metrics (i.e., CI and HI). This lower conformity could be explained by considering the fact that the optimization objectives and the normalization technique were kept the same with AAA plans. In other words, lower conformity does not indicate the AXB plans’ inferiority. In the case of PTVtis, though, the two algorithms provided a similar CI, which also confirms the theory that AAA has high calculation accuracy in homogeneous soft tissue media.

Lung doses calculated by AAA and AXB, i.e., MLD and V_20_, were also dependent on the PTV position and the density of the included target. Except for a few cases for PTVint, AAA overestimated both MLD and V_20_ by a mean of -5.5 to -30.7%, with the largest differences seen in PTVair. The lung results do not come to agreement with previously published works, but we should consider the different PTV locations and density. PTVint, which was the PTV with the most variable lung position, showed no statistical significance for either lung or spinal canal dose metrics. Contributing to that conclusion, Kroon et al. reported that AAA can over- or underestimate the lung dose depending on the actual combination of target location and lung density [1]. Notably, all lung doses were much lower than the QUANTEC constraints. The spinal canal dosimetry showed that AAA predicted higher maximum doses, similar to other studies [12,22], and we should also note here that PTVint showed the largest standard deviation in the dose metrics.

For the plans with different PTV margins, the plan with a 0.5 cm margin showed, in most cases, the worst CI and HI, as indicated in Figure 10. This can be attributed to the small PTV volume, i.e., 15 cc, which makes it difficult to deposit the dose uniformly, while taking into account that the linear accelerator multi-leaf collimator consists of 0.5 cm leaf widths. As the margin was expanded (i.e., 0.7, 1, and 1.5 cm), the CI and HI improved for both algorithms, indicating that larger volumes and small interface distances produce more uniform plans with better PTV coverage. Nevertheless, expanding the PTV margin also increases the MLD and V_20_ of the lungs, which are the main OAR, as well as the D_1%_ of the spinal canal. Therefore, attention is required in lung therapies, particularly in treatments with conventional dose fractionation where margins exceed 1 cm (GTV-PTV margin) [24,25]. For early-stage lung tumors treated stereotactically, higher doses of radiation per fraction are delivered, and PTV margins are smaller. Bissonnette et al. [24] reported a 5 mm margin to the ITV, confirmed by their setup margin study, which showed that a tolerance level of 5 mm reached a compliance level of 95%.

For all margins, MLD and lung V_20_ were lower for AXB plans. Additionally, analyzing the dosimetric results for the spinal canal, we could notice that AXB provided a lower maximum dose (D_1%_), as in the case of the PTVs in three different lung positions. The largest differences were found for the PTV 0.7 for both OARs, as well as for PTV D_max_, D_mean_, and D_min_. This was a noteworthy outcome, which led us to the following conclusion: for narrow margins (margin = 0.5 cm), neither algorithm could produce conformal plans. For larger ones (margins >0.7 cm), where the interface distance became smaller and the PTV could even include part of the tissue /bone surface (e.g., PTV 1.5), both algorithms showed more similar computational efficiency.

The MUs showed a decrease in the number of MUs for all the AXB plans. That can be explained by the fact that, keeping the same optimization objectives and normalization technique, AXB plans had much lower PTV dose coverage. Indeed, for PTVtis where the algorithms showed similar behavior, the difference in MUs was the smallest. On the other hand, for PTVair, where we had the largest dose differences, MUs had the largest standard deviation value. For clinical plans with different optimization objectives and acceptable dose coverage, the MU effect remains to be investigated. 

With regard to the limitations of the study, a larger sample would have been used to obtain more robust results for all the PTV categories. Additionally, a further study including AXB plans, which would have been optimized with optimization objectives suitable for achieving acceptable clinical plans, could provide a wider overview of the overall clinical impact of both algorithms in the treatment planning procedure. Finally, a patient-specific quality assurance (PSQA) procedure would have been applied to evaluate the effect of the two algorithms on the treatment plans’ overall quality [12,23]. Specifically, PSQA with the ArcCHECK (Sun Nuclear Corporation, FL-USA) phantom, available at our department, could be performed to obtain the gamma passing rate of each plan.

## Conclusions

With a simple-to-apply method, we investigated the dosimetric impact of the algorithms AXB and AAA in lung cancer treatments when changing the PTV location, margins, and interface distances. The results showed that the dose differences in PTV and OARs between AAA and AXB could be remarkable, especially for PTVs including much air volume and are relatively close to an air/tissue interface. AXB, which has shown accuracy comparable to MC methods, resulted in lower PTV conformity, lower OAR doses, and lower MU values. Therefore, special attention is required in clinical situations when implementing AAA for heterogeneous media and close to the interface PTVs. The quick and simple method presented here can be useful to confirm the appropriateness of implementing the AXB algorithm for lung treatments. The study’s limitations should be taken into consideration for the generalization of the results.

## References

[1] Kroon PS, Hol S, Essers M (2013). Dosimetric accuracy and clinical quality of Acuros XB and AAA dose calculation algorithm for stereotactic and conventional lung volumetric modulated arc therapy plans. Radiat Oncol.

[2] Fogliata A, Nicolini G, Clivio A, Vanetti E, Cozzi L (2012). Critical appraisal of Acuros XB and Anisotropic Analytic Algorithm dose calculation in advanced non-small-cell lung cancer treatments. Int J Radiat Oncol Biol Phys.

[3] Elcim Y, Dirican B, Yavas O (2018). Dosimetric comparison of pencil beam and Monte Carlo algorithms in conformal lung radiotherapy. J Appl Clin Med Phys.

[4] Baskar R, Lee KA, Yeo R, Yeoh KW (2012). Cancer and radiation therapy: current advances and future directions. Int J Med Sci.

[5] Pan Y, Fu Y, Zeng Y (2022). The key to immunotherapy: how to choose better therapeutic biomarkers for patients with non-small cell lung cancer. Biomark Res.

[6] Reynaert N, van der Marck S, Schaart D (2006). Monte Carlo Treatment Planning: An Introduction. Report 16 of the Netherlands Commission on Radiation Dosimetry (NCS). June.

[7] Van Esch A, Tillikainen L, Pyykkonen J (2006). Testing of the analytical anisotropic algorithm for photon dose calculation. Med Phys.

[8] Cheung ML, Kan MW, Yeung VT, Poon DM, Kam MK, Lee LK, Chan AT (2021). The effect on tumour control probability of using AXB algorithm in replacement of AAA for SBRT of hepatocellular carcinoma located at lung-liver boundary region. BJR Open.

[9] Fogliata A, Nicolini G, Vanetti E, Clivio A, Cozzi L (2006). Dosimetric validation of the anisotropic analytical algorithm for photon dose calculation: fundamental characterization in water. Phys Med Biol.

[10] Fogliata A, Nicolini G, Clivio A, Vanetti E, Mancosu P, Cozzi L (2011). Dosimetric validation of the Acuros XB Advanced Dose Calculation algorithm: fundamental characterization in water. Phys Med Biol.

[11] Vassiliev ON, Wareing TA, McGhee J, Failla G, Salehpour MR, Mourtada F (2010). Validation of a new grid-based Boltzmann equation solver for dose calculation in radiotherapy with photon beams. Phys Med Biol.

[12] Srivastava RP, Basta K, De Gersem W, De Wagter C (2021). A comparative analysis of Acuros XB and the analytical anisotropic algorithm for volumetric modulation arc therapy. Rep Pract Oncol Radiother.

[13] Huang B, Wu L, Lin P, Chen C (2015). Dose calculation of Acuros XB and Anisotropic Analytical Algorithm in lung stereotactic body radiotherapy treatment with flattening filter free beams and the potential role of calculation grid size. Radiat Oncol.

[14] Brennan SM, Thirion P, Buckney S, Shea CO, Armstrong J (2010). Factors influencing conformity index in radiotherapy for non-small cell lung cancer. Med Dosim.

[15] Landberg T, Chavaudra J, Dobbs J (1999). ICRU Report 62: Prescribing, Recording and Reporting Photon Beam Therapy (Supplement to ICRU Report 50). J ICRU.

[16] Bradley JD, Paulus R, Komaki R (2015). Standard-dose versus high-dose conformal radiotherapy with concurrent and consolidation carboplatin plus paclitaxel with or without cetuximab for patients with stage IIIA or IIIB non-small-cell lung cancer (RTOG 0617): a randomised, two-by-two factorial phase 3 study. Lancet Oncol.

[17] Kataria T, Sharma K, Subramani V, Karrthick KP, Bisht SS (2012). Homogeneity Index: an objective tool for assessment of conformal radiation treatments. J Med Phys.

[18] Lauve A, Morris M, Schmidt-Ullrich R (2004). Simultaneous integrated boost intensity-modulated radiotherapy for locally advanced head-and-neck squamous cell carcinomas: II--clinical results. Int J Radiat Oncol Biol Phys.

[19] Wyckoff HO (1976). Preface Reports of the International Commission on Radiation Units and Measurements. J ICRU.

[20] Han T, Followill D, Mikell J (2013). Dosimetric impact of Acuros XB deterministic radiation transport algorithm for heterogeneous dose calculation in lung cancer. Med Phys.

[21] Bush K, Gagne IM, Zavgorodni S, Ansbacher W, Beckham W (2011). Dosimetric validation of Acuros XB with Monte Carlo methods for photon dose calculations. Med Phys.

[22] Fleming C, O'Keeffe S, McDermott R, Dunne M, McClean B, León Vintró L (2021). The influence of Acuros XB on dose volume histogram metrics and tumour control probability modelling in locally advanced non-small cell lung cancer. Phys Med.

[23] Tsimpoukelli M, Patatoukas G, Chalkia M (2023). Dosimetric comparison and evaluation of two computational algorithms in VMAT treatment plans. J Appl Clin Med Phys.

[24] Bissonnette JP, Purdie TG, Higgins JA, Li W, Bezjak A (2009). Cone-beam computed tomographic image guidance for lung cancer radiation therapy. Int J Radiat Oncol Biol Phys.

[25] Nestle U, De Ruysscher D, Ricardi U (2018). ESTRO ACROP guidelines for target volume definition in the treatment of locally advanced non-small cell lung cancer. Radiother Oncol.

